# Dentofacial characteristics of children and adolescents with foetal alcohol spectrum disorders: a comparison with matched controls

**DOI:** 10.1186/s40510-023-00497-w

**Published:** 2023-12-26

**Authors:** Katarzyna Ludwików, Anna Westerlund, Nameer Al-Taai, Małgorzata Zadurska, Ewa Monika Czochrowska

**Affiliations:** 1 Department of Orthodontics, Medical University of Warsaw, ul. Binieckiego 6, 02-097 Warsaw, Poland; 2 Department of Orthodontics, Institute of Odontology, Sahlgrenska Academy, University in Gothenburg, Gothenburg, Sweden; 3 Department of Odontology, Umeå University, Umeå, Sweden; 4 Hamdan Bin Mohammed College of Dental Medicine, Mohammed Bin Rashid University of Medicine and Health Sciences, Dubai, United Arab Emirates

**Keywords:** Cephalometric analysis, Dentofacial characteristics, Foetal alcohol syndrome, Foetal alcohol spectrum disorders, Prenatal alcohol exposure

## Abstract

**Background:**

Foetal alcohol spectrum disorders (FASD) include somatic and neurological developmental disturbances after prenatal alcohol exposure, including facial anomalies. However, the knowledge of the orthodontic skeletal and dental cephalometric relations in this group is limited. The aim of the study was to assess the dentofacial characteristics of children and adolescents with FASD and to compare them with a matched control group.

**Methods:**

The study group comprised all available children and adolescents diagnosed with FASD (> 7 years of age) in whom good quality cephalograms were available. The control group comprised non-syndromic, orthodontically untreated children with normal occlusion and skeletal relations matched with age and gender. Cephalometric analysis included eighteen linear and angular measurements. The general linear model for repeated measures regarding age, gender and the type of FASD was applied.

**Results:**

The group with FASD included 35 individuals (21 girls and 14 boys) aged 7–18 years including 21 with foetal alcohol syndrome. The mean age in the study and the control group was 12.8 years (SD, range 3.2, 7.1–18.1) and 13.0 (SD, range 2.9, 9.1–18.1), respectively. Statistically significant differences between the groups were found in 15 out of 18 of the cephalometric measurements (83%). In children with FASD the mandible was more retrusive, the incisors were more proclined and the mandibular incisors and the lips were more protruded when compared with controls. There was no significant evidence of an influence of age, gender or FASD type.

**Conclusions:**

Dentofacial characteristics of children and adolescents with FASD significantly differ from controls. Early orthodontic diagnosis and prophylaxis should play a part of the interdisciplinary care of patients in this group.

## Introduction

Foetal alcohol spectrum disorders (FASD) relate to a complex, untreatable syndrome of developmental defects resulting from prenatal exposure to alcohol [1]. Lemoine et al. were the first to report on the impact of alcohol consumption on the foetus in 1968 [2], while Jones et al. in 1973 described associated dysmorphic facial features and defined them as the foetal alcohol syndrome (FAS), which is the most severe form of FASD [3]. The FASD comprises a broader diagnosis and includes individuals both with FAS and others who do not meet all criteria for FAS but are affected by the prenatal alcohol exposure. This includes neurodevelopmental and neurobehavioral disorders and problems with various organs such as heart and kidneys, also vision and hearing defects. The global prevalence of FASD among children and adolescents in the general population was estimated at 7.7 per population of 1000. The WHO European Region had the highest prevalence (19.8 per 1000), and the WHO Eastern Mediterranean Region had the lowest (0.1 per 1000). Of 187 countries, South Africa was estimated to have the highest prevalence of FASD at 111.1 per 1000 population, followed by Croatia at 53.3 and Ireland at 47.5 [4]. In Poland, the incidence of FASD is at ≥ 20 cases per 1000, and FAS at 4 per 1000 [5]. In recent years, an increased tendency of women to consume alcohol has been observed, most notably at the age of 18–29 years, which may as a consequence increase the incidence of children born with FASD. It is important to note that early diagnosis of developmental disorders in children with FASD will improve the odds for the therapy and the support system to be effective, and that also concerns the institutional caregivers.

The diagnostic protocol of FASD is complex and interdisciplinary due to the multitude of symptoms. In Poland, since 2012 the Washington 4-Digit Diagnostic Code, compiled in 2004 by the researchers from Seattle, has been widely applied [6]. It comprises five key areas of FASD, in the following order:

1. Prenatal alcohol exposure
2. Insufficient weight and height
3. Craniofacial irregularities including small cranial circumference, short neck, low, asymmetric position of the ears, deformed auricles, narrow forehead, retrognathia and retrogenia, flat midface, narrow palpebral fissures, eyes farther apart than normal, epicanthal fold, short and small nose, long upper lip with narrow vermilion border, flattened or absent midline groove in the philtrum
4. Damage to the central nervous system
5. Skeletal and circulatory developmental disorders

Despite a number of publications on FASD in the international medical journals, including dental research studies, only few pertain to the orthodontic characteristics of children with FASD; the description of a standard protocol for orthodontic treatment is also absent.

Recently performed clinical dental and orthodontic evaluation of children and adolescents with FASD confirmed increased prevalence of dysfunctions and parafunctions such as mouth breathing, thumb sucking and nail biting [7]. Distal occlusion, the presence of crossbites and borderline need for orthodontic treatment were more common in the FASD group compared to the general population. So far, the orthodontic description of the skeletal and dental relations in individuals with FASD is limited. Naidoo et al. demonstrated that children with FAS, when compared with non-syndromic controls, presented with vertically and horizontally underdeveloped maxillae, together with features of a long-face syndrome with a large gonial angle and a short ramus in relation to the total face height [8]. The study group included ninety ‘coloured’ children from South Africa with the mean age of 8.9 years who were compared with ninety control children matched for age, gender and social class, but no information on the type of occlusion was provided. In another study, in which lateral cephalometric radiographs were quantitatively assessed in a series of fifteen American black children with FAS and compared to the matched controls, but also no information on the type of occlusion was provided. The findings disclosed a triad of facial profile differences: (1) frontal bossing, (2) palatal plane tipped up in the front with proclined upper incisors and a acute nasolabial angle, and (3) above-average length of the mandibular corpus [9]. Interestingly, they have found that the midface was unremarkable in size and position when compared to the control children. Another analysis of cephalometric radiographs of twelve children with FAS corroborated the clinical observation of midfacial deficiency which was related to the retrusion of the maxilla confirmed in the study group [10]. In these studies, cephalometric analysis was performed only for children with FAS but the maxilla–mandible relationship with subdivisions was not examined.

The aim of the study was to assess the dentofacial characteristics of children and adolescents with FASD and to compare them with a matched, non-FASD, control group with normal occlusion and skeletal relations.

## Material and methods

### Study group

All available individuals who were diagnosed with the foetal alcohol spectrum disorders (FASD) were enrolled. The study sample was collected with the help offered by foundations dealing with disabled children, FASD diagnostic facilities, associations of adoptive and foster families, and child care homes. Participation in training courses and support groups meetings for families with children affected by FASD, as well as announcements about this particular study in social media, was another way to obtain willing participants.

All the children included in the study had a written diagnosis confirming the presence of FASD. As well as the most severe manifestation which is FAS, the FASD also includes Foetal Alcohol Effects (FAE), which is FAS without visible external deformities, Partial Foetal Alcohol Syndrome (pFAS) (the presence of several physical and neurological features characteristic of FAS), Alcohol-Related Birth Defect (ARBD) (which identifies physical anomalies resulting from confirmed exposure to alcohol), Alcohol-Related Neurodevelopmental Disorders (ARND)—neurological disorders related the effect of alcohol, and Foetal Alcohol-Related Conditions (FARC).

### Control group

The control group included non-syndromic children and adolescents with normal occlusion and normal skeletal (vertical and sagittal) relations matched one-to-one on age and gender with the FASD sample from the files of the Orthodontics, Department of Odontology Umeå University, Sweden. None of the controls had been orthodontically treated [11].

### Cephalometric examination

Digital cephalometric radiographs were taken using a cephalostat in a MyRay Hyperion X6Pro, Kodak 9000, with an object-to-film distance of 15 cm, 8 mAs exposure (0.8 s 10 mA current) and a 66–70 kVp. They were manually digitized using a computer software WebCeph Image™ with 18 measurements (5 linear and 13 angular). Description of the landmarks, planes and measurements is given in Tables 1 and 2. These 15 variables allowed assessment of size, shape, and relative position of three craniofacial complexes: (1) the cranial base, (2) midface, and (3) mandible. In addition, 3 variables were computed to compare soft tissue profiles.

**Table 1:** Abbreviations for the anatomical landmarks and reference planes used in the cephalometric analysis

Landmark/plane	Description
S	Sella turcica
N	Nasion
A	Subspinale
B	Supramentale
Pg	Pogonion
ANS	Anterior nasal spine
PNS	Posterior nasal spine
Go	Gonion
Gn	Gnathion (anatomical)
Ar	Articulare
U1R	Upper central incisor root tip
L1R	Lower central incisor root tip
U1T	Upper central incisor incisal tip
L1T	Lower central incisor incisal tip
ULP	Upper lip point
LLP	Lower lip point
ProN	Pronasale
Col	Columella
ST Pog	Soft tissue pogonion
SN	Sella-Nasion plane
ANS-PNS	Maxillary plane
Go-Gn	Mandibular plane
E-plane	Extends from the tip of soft tissue nose to the chin (Rickett’s aesthetic plane)

**Table 2:** Measurements and definitions used in the cephalometric analysis

Measurement (unit)	Description
SNA (°)	Sagittal position of the maxilla
SNPog (°)	Sagittal position of the chin
SNB (°)	Sagittal position of the mandible
ANPog (°)	Sagittal chin relation
ANB (°)	Sagittal jaw relations
SN-ANS-PNS (°)	Maxillary inclination in relation to the SN plane
SN-Go-Gn (°)	Mandibular inclination in relation to the SN plane
ANS-PNS-Go-Gn (°)	Angle between the maxillary and the mandibular planes
Gonial angle (Ar-Go-Gn) (°)	Angle of the mandible
U1-ANS-PNS (°)	Angle formed by the long axis of the upper incisor and the maxillary plane
L1-Go-Gn (°)	Angle formed by the long axis of the lower incisor and the mandibular plane
L1-APog (mm)	Distance from the lower incisor incisal tip to the A-Pog line
Overjet (mm)	Distance from the incisal tip of upper incisors and the incisal tip of the lower incisors in the horizontal direction
Overbite (mm)	Distance from the incisal tip of upper incisors and the incisal tip of the lower incisors in the vertical direction
Interincisal angle (°)	Angle between the long axes of the upper incisor and the lower incisor
Nasolabial angle (°)	Angle formed by the two lines passing through the lower tip of the nose (the columella) and the tip of the upper lip
Upper lip to E-plane (mm)	Distance from the upper lip to the E-plane
Lower lip to E-plane (mm)	Distance from the lower lip to the E-plane

All cephalometric measurements were performed twice within 4-week interval by the same operator (K.L.) and reliability was assessed by intra-class correlation coefficients. When discrepancies in landmark identification were observed, a second examiner (E.Cz.) independently evaluated the tracings, and corrections were made. The mean error and 95% confidence interval (CI) between the repeated records were calculated as follows: 0.8 mm (0.5–0.9 mm) for linear measurements and 0.7° (0.6°–0.8°) for angular measurements; reliability coefficient (r) ranged from 92 to 98% and from 94 to 98%, respectively.

### Statistical method

Standard descriptive statistics tools were used to describe the material. The differences between measurements in the FASD and control groups (diff = FASD – Control) were analysed as a main endpoint. The main effect was tested using the general linear model (GLM) for repeated measures regarding age, gender and the type of FASD (FAS, pFAS, ARND) as the between subject variables. The age variable was categorized: age for two categories: 0: <  = 12 and 1: > 12. The FASD type variable was encoded as 0: ARND, 1: FAS, 2: pFAS. The statistical significance of the differences for individual measurements was tested using the student's T test. All statistical analyses were performed using IBM SPSS Statistics 23 package.

## Results

The study group comprised 35 Caucasian children and adolescents (21 girls and 14 boys) with the mean age of 12.8 years (SD, range 3.2, 7.1–18.1 years) (Table 3). All the children in the study group resided in foster families, child care homes or had been adopted. None of the children had other dentofacial deformities like clefts or other syndromes and none had been orthodontically treated. The examined children mostly came from Pomeranian, Masovian and Kuyavian-Pomeranian Provinces, accounting for approximately 30% of the Polish population. The control group included 35 participants. The control group included 35 children with the mean age of 13.0 years (SD, range 2.9, 9.1–18.1). There was no significant evidence of the effect of age and gender between the study and the controls groups.

**Table 3:** The characteristics of the study group in relation to different FASD types

Diagnosis	FASD	FAS	pFAS	ARND
Number of children (%)	35 (100%)	21 (60%)	7 (20%)	7 (20%)
*Age (years)*				
Range	7.1 – 18.1	7.4 − 17.9	8.4 − 15.7	7.1 − 18.1
Mean	12.8	12.9	12.6	12.6
*Gender*				
Girls	21	15	2	4
Boys	14	6	5	3

The study group included 21 children and adolescents diagnosed with FAS, 7 with pFAS and 7 with ARND and the characteristics of the group are presented in Table 3.

**Fig. 1:**
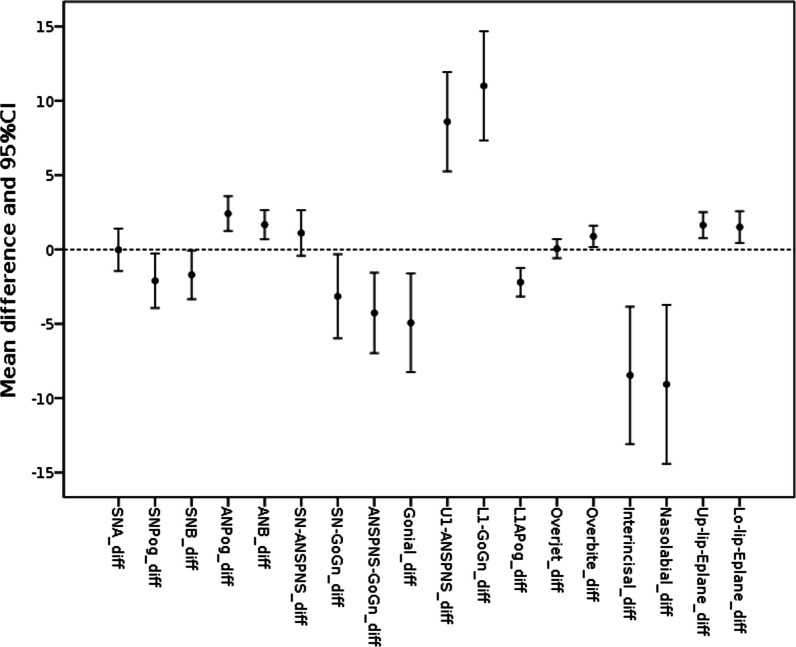
Differences between the FASD and the control group in the cephalometric measurements

The description of the cephalometric measurements for the FASD and control groups is presented in Table 4. The norms are also given. In Table 5, the differences between the FASD and the control groups regarding the cephalometric examination are summarized. For 15 of the 18 (83%) of the cephalometric measurements (SNPog, SNB, ANPog, ANB, SN-Go-Gn, ANS-PNS-Go-Gn, Gonial angle, U1-ANS-PNS, L1-Go-Gn, L1-APog, Overbite, Interincisal angle, Nasolabial angle, Upper lip-E-plane, Lower lip-E-plane) statistically significant differences were found (*p* < 0.05). Negative difference values indicate that the measurement in the FASD group was smaller than in the control group and positive values indicate the opposite, which is also shown in Fig. 1.

**Table 4:** Descriptive statistics for the cephalometric measurements in the FASD and control groups

Measurement	FASD				Control				Norm
	Min	Max	Mean	SE	Min	Max	Mean	SE	
SNA	74.7	88.9	81.7	0.61	74.2	87.4	81.7	0.51	82 ± 3.5
SNPog	70.9	89.0	78.5	0.73	74.1	84.8	80.6	0.52	80 ± 3.5
SNB	70.3	88.5	77.8	0.68	73.5	84.0	79.5	0.49	80 ± 3
ANPog	0.28	9.57	3.47	0.39	− 4.50	3.90	1.05	0.35	2 ± 2.5
ANB	− 0.27	9.14	3.86	0.40	− 1.90	4.70	2.19	0.30	2 ± 2
SN-ANS-PNS	0.62	15.8	7.97	0.67	0.90	10.5	6.87	0.48	8 ± 3
SN-Go-Gn	18.7	45.1	29.1	1.12	25.6	39.7	32.3	0.62	33 ± 2.5
ANS-PNS-Go-Gn	11.7	33.9	21.2	1.00	17.9	33.2	25.4	0.71	25 ± 6
Gonial angle	109	138	123	1.20	120.4	140	128	0.91	127 ± 8
U1-ANS-PNS	92.3	126	112	1.14	88.6	115	103	1.24	110 ± 6
L1-Go-Gn	78.0	114	98.8	1.31	79.6	102	87.8	1.01	94 ± 7
L1-APog	− 1.76	7.91	1.86	0.37	0.80	8.50	4.07	0.31	2 ± 2
Overjet	− 2.15	9.68	3.82	0.34	1.80	5.40	3.76	0.19	3.5 ± 2.5
Overbite	− 0.16	5.69	3.23	0.27	− 1.60	4.90	2.35	0.32	2 ± 2.5
Interincisal angle	105	147	128	1.54	114	152	136	1.68	132 ± 6
Nasolabial angle	70.0	124	105	2.01	96.8	134	114	1.66	110 ± 7
Upper lip-E-plane	− 6.92	2.10	− 1.59	0.36	− 7.20	0.80	− 3.22	0.35	0.5 ± 2.5
Lower lip-E-plane	− 5.48	4.64	− 0.35	0.38	− 4.70	2.70	− 1.85	0.40	0.5 ± 1.5

**Table 5:** Differences between the FASD and the control groups in the cephalometric measurements

Measurement	Difference (FASD—Control)					
	Min	Max	Mean	95%CI		*p* value
SNA	− 6.73	9.60	− .027	− 1.46	1.40	.970
SNPog	− 12.5	7.91	− 2.10	− 3.93	− .268	.026
SNB	− 10.0	8.96	− 1.71	− 3.34	− .072	.041
ANPog	− 2.15	11.8	2.41	1.249	3.58	.000
ANB	− 3.71	8.62	1.67	.681	2.65	.002
SN-ANS-PNS	− 8.19	7.61	1.11	− .426	2.64	.151
SN-Go-Gn	− 17.6	19.5	− 3.15	− 5.98	− .324	.030
ANS-PNS-Go-Gn	− 17.9	14.8	− 4.27	− 7.00	− 1.57	.003
Gonial	− 27.1	16.4	− 4.93	− 8.25	− 1.62	.005
U1-ANS-PNS	− 9.05	31.1	8.59	5.26	11.9	.000
L1-Go-Gn	− 16.6	30.9	11.0	7.33	14.7	.000
L1-APog	− 7.46	3.11	− 2.21	− 3.17	− 1.25	.000
Overjet	− 4.25	4.78	.053	− .589	.695	.867
Overbite	− 3.15	4.30	.886	.167	1.61	.017
InterIncisal	− 40.6	16.5	− 8.47	− 13.1	− 3.85	.001
Nasolabial	− 49.8	22.1	− 9.07	− 14.4	− 3.73	.002
Upper lip-Eplane	− 2.83	7.90	1.63	.761	2.51	.001
Lower lip-Eplane	− 4.90	9.34	1.51	.438	2.57	.007

The SNPog and SNB angles were significantly smaller in the FASD group indicating more retrusive position of the mandible in these children. There was no significant difference in the SNA angle between the groups, while the ANPog and ANB angles were significantly bigger in the study group which confirmed more distal jaw relations in children with FASD. The maxillary inclination did not significantly differ between the groups (SN-ANS-PNS), while the mandibular inclination (SN-Go-Gn) was smaller in the study group than in controls. Also, the ANS-PNS, Go-Gn and Gonial angles were significantly smaller in the FADS group. The maxillary and mandibular incisors were significantly more proclined, and the mandibular incisors were less protruded in children with FASD than in the controls. Also, the overbite was significantly increased in the study group, while the overjet did not differ between the groups. The interincisal angle and the nasolabial angle were significantly smaller in children with FASD, while the upper and the lower lips were significantly more protruded.

**Fig. 2:**
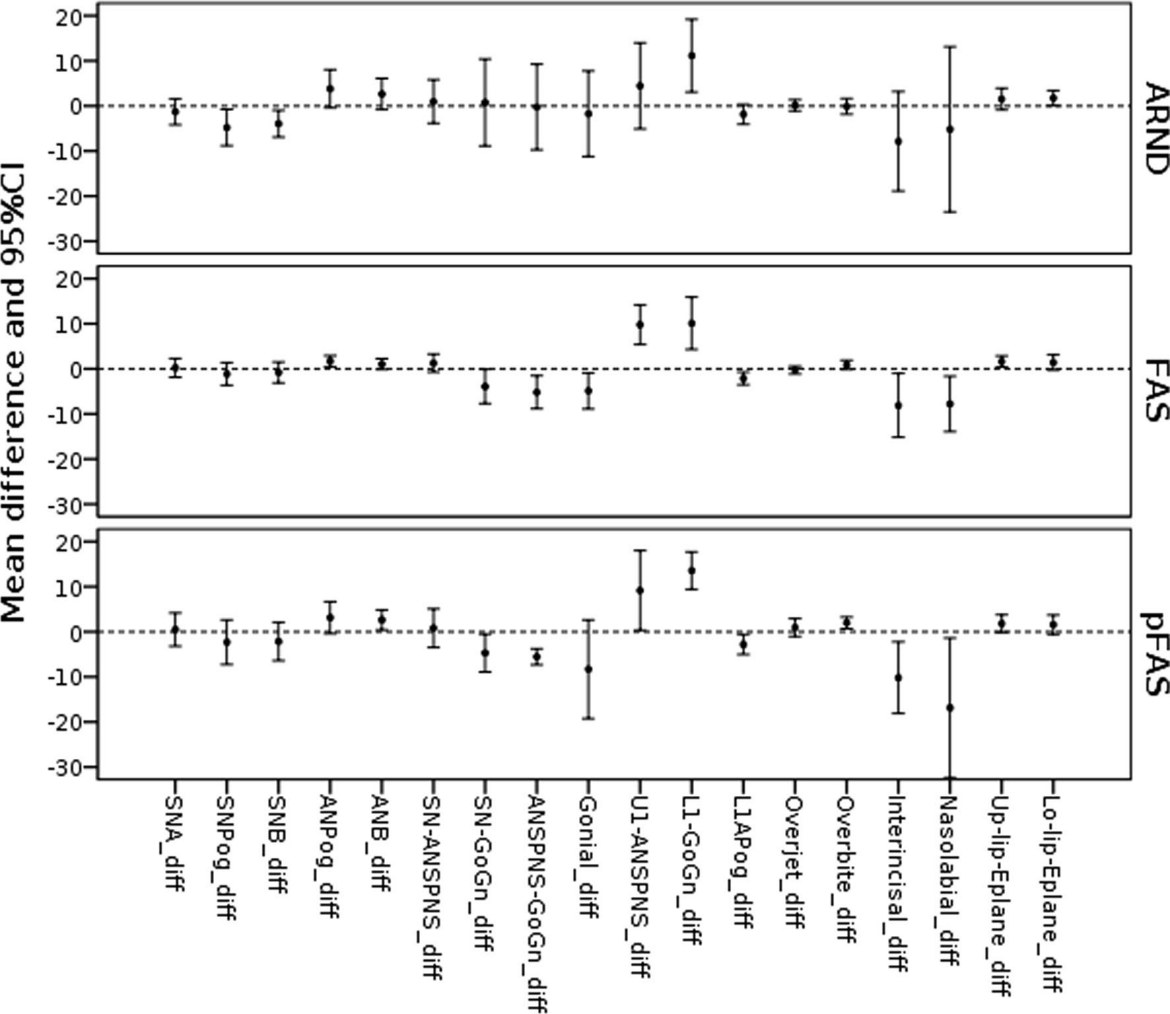
Cephalometric measurements differences in relation to the FASD type

There was no significant evidence of the effect of the FASD subgroup type (FAS, pFAS, ARND) between the study and the controls groups on the difference in the cephalometric measurements (*p* > 0.1) (Table 6, Fig. 2). Also, there were no significant differences between all children with FAS and pFAS and children with ARND in any of the cephalometric measurements.

**Table 6:** Means and standard deviations differences in relation to the FASD type

Measurement	FAS		PFAS		ARND	
	Mean	SE	Mean	SE	Mean	SE
SNA	0.23	1.00	.049	1.50	− 1.32	1.18
SNPog	− 1.14	1.21	− 2.31	2.02	− 4.79	1.65
SNB	− 0.81	1.12	− 2.15	1.74	− 3.95	1.21
ANPog	1.71	0.59	3.14	1.45	3.80	1.72
ANB	1.03	0.58	2.62	0.90	2.63	1.41
SN-ANS-PNS	1.26	0.96	0.80	1.74	0.97	1.97
SN-Go-Gn	− 3.92	1.82	− 4.72	1.72	0.73	3.93
ANS-PNS-Go-Gn	− 5.18	1.76	− 5.55	0.73	− 0.25	3.88
Gonial angle	− 4.88	1.92	− 8.30	4.47	− 1.74	3.88
U1-ANS-PNS	9.80	2.11	9.15	3.60	4.43	3.90
L1-Go-Gn	10.1	2.78	13.6	1.69	11.14	3.30
L1-APog	− 2.13	0.68	− 2.80	0.91	− 1.86	0.89
Overjet	− .026	0.42	0.94	0.81	0.11	0.51
Overbite	0.86	0.49	1.96	0.53	− 0.11	0.71
InterIncisal	− 8.10	3.39	− 10.2	3.25	− 7.85	4.50
Nasolabial	− 7.77	2.94	− 16.7	6.34	− 5.17	7.48
Upper lip-Eplane	1.61	0.60	1.82	0.79	1.52	0.98
Lower lip-Eplane	1.40	0.81	1.58	0.87	1.75	0.69

## Discussion

The study revealed significant differences in cephalometric measurements applied to orthodontic diagnostics that pertain to maxilla–mandible relationship, incisor inclination and position and soft tissue profile between children with FASD and the matched untreated control children with normal occlusion. So far, such cephalometric comparisons were not reported in the literature. The previous studies included children with FAS only and did not report occlusal status of the control children (8–10). The study group comprised 35 children with FASD including 21 children with FAS with the mean age of 12.8 years, which constitutes one of the most numerous study groups reported in the literature subjected to a cephalometric examination. To collect a representative sample of children diagnosed with FASD in whom cephalometric radiographs could be taken is challenging, since these children are usually subject to institutional care such as foster or adoptive families and are scattered all over the country.

### FASD classification

All the children covered by the study submitted a written diagnosis confirming the presence of FASD, i.e. the 2004 4-digit with Diagnostic Questionnaire from Seattle and the 2005 Canadian Questionnaire. In 2017, Astley et al. demonstrated that the two systems showed similarities with respect to application of precise criteria and diagnosing the whole spectrum of consequences of prenatal exposure to alcohol [11]. Increased interest in FASD among Polish specialists and the absence of uniform management protocol that could be followed in diagnostic centres prompted the introduction of guidelines to diagnose the FASD spectrum. They were put forward by a team of interdisciplinary Polish scientists in 2020 on the initiative of the National Agency for the Prevention of Alcohol-Related Problems (PARPA). Since then, two types of medical disorders labelled as FASD have been distinguished:

- FAS (in ICD‐10 classification coded Q86.0).
- ND‐PAE (neuro‐developmental disorders associated with prenatal alcohol exposure)—and a non-diagnostic category, namely the risk of FASD [12].

The recruitment of children for this study commenced before 2020, therefore, a former classification of FASD was used in this study. It can be argued that children with diagnosed ARND today could be classified as ND-PAE, but re-taking diagnostic examinations (cephalometric measurements) was not possible because the children’s places of residence were dispersed and concerned different care institutions. No significant differences were observed in relations to the FASD subgroup type, but pFAS and ARND groups were less numerous than the group with FAS.

### Skeletal characteristics

Significant skeletal differences included more retrusive position of the mandible and therefore more distal jaw relations in children with FASD compared with children with normal occlusion. However, the maxillary position did not significantly differ between the groups. This is interesting to note because retrognathia was considered common in children with FASD. The mean age in the study group was 12.8 years, which means that anteroposterior growth of the maxilla was generally completed in most of the subjects with FASD and the maxillary position was not more retrusive compared to the population with normal occlusion. The same results were also obtained for the children with FAS when compared with controls but the mean difference was the smallest in relation to two other subgroups. Shortening of the anterior cranial base, which was reported in children with FAS, can result in the increase of SNA angle values, and hence lack of significant differences with the control children.

The SN-Go-Gn and the gonial angles are significantly smaller in the FASD group, which indicates a tendency for a more horizontal inclination of the mandible and a reduced angle between the mandibular ramus and the mandibular body. Reduced gonial angle is the most frequently seen skeletal factor signifying the importance of angulation and growth of ramus in development of deep bite [13]. This is at variance with the findings of Naidoo et al. who reported that the children with FAS presented with vertically and horizontally underdeveloped maxillae, while the mandible was in a normal anteroposterior and sagittal position and had a shorter body, a shorter ramus but a slightly larger gonial angle [8]. Frias et al. observed midfacial deficiency that this abnormality was not caused by true maxillary hypoplasia but by retrusion of the maxilla. They postulated that this abnormal position was secondary to restricted forward growth of the face resulting from abnormal brain growth and subsequent shortening of the anterior cranial base [10]. More retrusive mandibular position and more distal jaw relations present in the study group corresponded well with the previously published results from the clinical examination of 67 children with FASD including 34 with FAS where more than half of children with FASD were diagnosed with Angle Class II [7]. In children with FAS this percentage was over sixty per cent.

### Dental characteristics

Significant dental differences included increased incisor proclination and more protruded lower incisor position in children with FASD. This may be related to a high incidence of parafunctions and muscular dysfunctions in children with FAS. Ludwików et al. reported more frequent occurrence of mouth breathing, thumb thrusting or nail biting in this group of children [7]. Children who breathe through the mouth and who rotate the mandible in a posterior and inferior direction may develop Class II malocclusions with increased overjet. In the present study, however, increased overjet was not observed when compared with the controls. Caregivers also reported frequent nail biting in children with FASD (41%). Nail biting is a typical stress-relieving habit. Nail biting may induce abrasions of the incisal edges of lower incisors and proclination of the upper incisors [14]. Similar observations were made in a study in which proclined upper incisors and a sharp nasolabial angle was acquired from thumb sucking [9]. Children with FASD had increased overbite in comparison with the controls. There was also a tendency for the development of an anterior open bite, which appears to be compensated for by an increase in the vertical dimension of the anterior alveolar process to bring the incisors into occlusion. The latter adaptation occurred mainly in the mandible [8].

### Profile evaluations

Main significant differences in soft tissue profile include reduced nasolabial angle and protruded lips in children with FASD in comparison with the control children. More obtuse nasolabial angle and protrusion of the upper lip is common in patients with distal malocclusions and the protrusion of the lower lip might be associated with a higher tendency for mouth breathing in this group. In another study, however, the nose, lips and facial proportions were found to be similar in the two groups, the one exception being the LI—Pogonion length which was significantly different [8]. Interestingly, no significant differences were found when cephalometric measurements were compared between three FASD subgroups and likewise when FAS and pFAS children were compared with those with ARND. This may indicate that developmental defects in the lower craniofacial region do not significantly differ between groups or generally are not much pronounced. However, the results of the study related to FASD subtypes should be interpreted with caution due to major differences in the sizes of the subgroup samples.

### General remarks on the dentofacial characteristics of children and adolescents with FASD

The results of this study may raise awareness of the occurrence of developmental disorders related to the dentofacial complex and the need for prompt interceptive or orthodontic treatment in this group of patients. None of the participants had been treated orthodontically, which may indicate low awareness of malocclusions in these children. On the other hand, the results of an earlier study confirmed a higher incidence of distal occlusion in children with FASD [7]. For this reason, institutional caregivers may overlook the problem especially if these children are not aware of smile aesthetics or orthodontic needs. Implementation of interceptive treatment at an early stage including also dysfunctions and parafunctions described in a previous study may considerably impact the normalization of craniofacial development and development of less severe skeletal malocclusions [7]. The increased frequency of dental and skeletal malocclusions present in the examined group children with FASD should be an important reason to include this group of developmental disorders in national health programmes, if not applicable.

### Limitations

Future studies should take into consideration changes in the FASD diagnostics and increase the sample size in particular for children and adolescents with FASD spectrum (non-FAS). The position of the maxilla in relation to the anterior cranial base should be further examined and compared with the control sample. The effect of the patients’ age on these measurements should also be investigated. All included children were older than 7 years of age; therefore, it would be beneficial to monitor earlier the development of the dentofacial complex in children with FASD with a longitudinal study in the future.

## Conclusions

Dentofacial characteristics of children and adolescents with FASD differ significantly from children with normal occlusion and skeletal relations. Children with FASD had a more retrusive position of the mandible and increased mandibular inclination, their incisors were significantly more proclined and the interincisal angle was smaller. The nasolabial angle was reduced with protruded upper and lower lips. Early orthodontic screening should be a part of the interdisciplinary care in children with FASD including management of oral dysfunctions and parafunctions.

## Data Availability

The datasets used and/or analysed during the current study are available from the corresponding author on reasonable request.

## Abbreviations

ARND: Alcohol-related neurodevelopmental disorders
FAS: Foetal alcohol syndrome
pFAS: Partial foetal alcohol syndrome
FASD: Foetal alcohol spectrum disorders
